# Using *C. elegans* to discover therapeutic compounds for ageing-associated neurodegenerative diseases

**DOI:** 10.1186/s13065-015-0143-y

**Published:** 2015-11-26

**Authors:** Xi Chen, Jeff W. Barclay, Robert D. Burgoyne, Alan Morgan

**Affiliations:** Department of Cellular and Molecular Physiology, Institute of Translational Medicine, University of Liverpool, Crown St, Liverpool, L69 3BX UK; Centre for Neurodegenerative Science, Van Andel Research Institute, 333 Bostwick Avenue NE, Grand Rapids, Michigan, MI 49503 USA

**Keywords:** Adult onset neuronal ceroid lipofuscinosis, Aging, Alzheimer’s disease, Amyotrophic lateral sclerosis, Caenorhabditis elegans, Compound screening, Frontotemporal dementia, Huntington’s disease, Neurodegeneration, Parkinson’s disease

## Abstract

Age-associated neurodegenerative disorders such as Alzheimer’s disease are a major public health challenge, due to the demographic increase in the proportion of older individuals in society. However, the relatively few currently approved drugs for these conditions provide only symptomatic relief. A major goal of neurodegeneration research is therefore to identify potential new therapeutic compounds that can slow or even reverse disease progression, either by impacting directly on the neurodegenerative process or by activating endogenous physiological neuroprotective mechanisms that decline with ageing. This requires model systems that can recapitulate key features of human neurodegenerative diseases that are also amenable to compound screening approaches. Mammalian models are very powerful, but are prohibitively expensive for high-throughput drug screens. Given the highly conserved neurological pathways between mammals and invertebrates, *Caenorhabditis elegans* has emerged as a powerful tool for neuroprotective compound screening. Here we describe how *C. elegans* has been used to model various human ageing-associated neurodegenerative diseases and provide an extensive list of compounds that have therapeutic activity in these worm models and so may have translational potential.

## Background

Despite decades of intense molecular research and the identification of many specific causative mutations, debilitating neurodegenerative diseases (NDs) including common disorders such as Alzheimer’s disease (AD) and Parkinson’s disease (PD), afflict millions worldwide and remain a significant and unresolved financial and social burden. Indeed, as ageing itself is by far the greatest risk factor for these diseases, this burden is set to increase dramatically as a result of our increasingly ageing population. Given the urgent need for therapies for these devastating and eventually fatal disorders, many researchers have developed animal models of NDs in order to screen for potential new drugs. In this review, we focus on compound screens performed in the nematode worm, *Caenorhabditis elegans*. We describe various different NDs that have been modelled in worms and list the therapeutic compounds that have been identified for each. In some cases, these compounds have also been shown to be protective in mammalian ND models, suggesting translational potential for human patients. We conclude that the combination of accurate genetic ND worm models with high-throughput automated drug screening platforms is a potentially very efficient strategy for early therapeutic drug discovery for NDs.

## Review

### An overview of human neurodegenerative diseases

NDs are characterised by progressive neuropsychiatric dysfunction and the loss of structure and function of specific neuronal circuitry that in turn result in behavioural symptoms. NDs can occur on a completely hereditary basis (e.g. Huntington’s disease), or can be hereditary and also appear sporadically in the majority of cases (e.g. AD, PD). In spite of the diversity in the underlying genes involved, inheritance patterns, clinical manifestation and exact sites of neuropathology, the rare, early onset familial (also known as Mendelian) forms and the more prevalent late-onset sporadic forms of different NDs share some common genetic origins and pathological hallmarks, such as the progressive and chronic nature of the disease, the extensive loss of specific neuronal subtypes, synaptic dysfunctions, the formation and deposition of misfolded protein aggregates [1–3]. Research and technological innovations over the past 10 years have made considerable progress in the elucidation of mechanisms of ND initiation and progression that lead to neurodegeneration. Emerging common themes in the pathogenesis of neurodegeneration include: aberrant phosphorylation, palmitoylation and acetylation of disease-causing proteins, protein misfolding, deficient ubiquitin–proteasome system (UPS) or autophagic process to clear disease-causing proteins, altered RNA metabolism, oxidative stress, mitochondrial dysfunction, excitotoxicity, disrupted axonal transport, neuroinflammation and microglial activation [4]. Linkage analysis, high-throughput sequencing and genome-wide association studies (GWAS) have also identified susceptibility genes in many NDs (Table 1) and promise to help unravel even more genes, novel loci and common genetic variants associated with the diverse collection of human NDs. Thus developments of therapeutic interventions that are applicable across the broad spectrum of NDs and target the shared pathogenic mechanisms may offer the best hope for a future neuroprotective therapy.

**Table 1:** A list of published *C. elegans* models of human neurodegenerative diseases and drugs that were shown to confer neuroprotection

NDs	Model	Strain/transgene name/(plasmid)	Expression in *C. elegans*	Phenotypes	Efficacious compounds identified/validated	References
Transgenic overexpression of human neurodegeneration-associated protein/peptide						
AD	P_*unc*-*54*_::*Aβ*_*1*–*42*_ (wild type); Dimer *Aβ*_*1*–*42*_ or Met^35^Cys *Aβ*_*1*–*42*_	CL2005, CL2006, CL1019, CL1118, CL1119, CL1120, CL1121, CL2120; CL2109, CL3109; CL3115	Constitutive muscles	Age-dependent progressive paralysis; forms amyloid deposits; increased oxidative stress CL2109, CL3109 and CL3115: no formation of amyloid deposits and no increase in oxidative stress	CL2006: caffeine, tannic acid and bacitracin; epigallocatechin gallate; reserpine; *Ginkgo biloba* extract EGb 761; soya isoflavone glycitein; oleuropein aglycone rifampicin; thioflavin T; curcumin; ferulic acid; fluoxetine; JWB1-84-1 and JAY2-22-33; NT219 CL2120: PBT2	[7, 28, 89–91]
	*dvIs100* [pCL354(*unc*-*54*:DA- *Aβ*_*1*–*42*_) + pCL26(*mtl*-*2*: GFP)].	GMC101		Severe and fully penetrant paralysis within 48 h after temperature shift	PBT2	[31]
	*smg*-*1(cc546);Is* [P_*myo*-*3*_::*Aβ*_*1*–*42*_::let UTR) + *(rol*-*6(su1006)*]	CL4176	Inducible body wall muscles	Rapid paralysis; oxidative stress precedes amyloid deposition; autophagosome accumulation	Coffee extracts, tetracycline and related analogs; copper; *Ginkgo biloba* extract EGb 761 and Ginkgolide A and J; Liuwei Dihuang (LWDH); galanthamine; icariside II; cocoa peptide; Carqueja (Baccharis trimera) oleuropein aglycone	[28, 92–95]
	*smg*-*1(cc546); Is*[P_*myo*-*3*_::GFP::degron + P_*mtl*-*2*_::GFP]	CL2337		Rapid paralysis; formation of stable perinuclear deposits		[96]
	*smg*-*1(cc546); Is*[P_*snb*-*1*_::*Aβ*_*1*-*42*_ + P_*mtl*-*2*_::GFP]	CL2241, CL2355	Inducible pan-neuronal	CL2241 exhibit WT movement. CL2355 is defective in chemotaxis toward benzaldehyde, associative learning, and thrashing in liquid; hypersensitive to serotonin; forms amyloid deposits; has partial sterility due to germline proliferation defects and embryonic lethality	CL2355: *Ginkgo biloba* extract EGb 761	[15, 96, 97]
	N2; *Is* [P_*eat*-*4*_::*ssAβ*_*1*-*42*_(N-terminus) + P_*eat*-*4*_::*gfp* + P_*myo*-*2*_::*mCherry*]	UA166	Glutamatergic neurons	Loss of GFP-marked glutamatergic neurons in an age-related manner; at day 3 only 48 % of worms had five intact glutamatergic neurons, and at day 7 only 25 % did	Clioquinol	[98]
	N2; *ynIs13*[P_*snb*-*1*_:APL-1] N2; *ynIs104*[P_*rab*-*3*_ *::apl*-*1*::GFP]	LGIII, LGV, LGX	Constitutive pan-neuronal	Defects in brood size, movement, and viability; severe chemotaxis defects and diminished touch habituation		[99, 100]
ALS	N2; *Is*[P_*hsp*-*16.2*_ *::SOD*-*1 (WT, A4* *V, G37R, G93A)* + P_*myo*-*3*_ *::SOD*-*1 (WT, A4* *V)*::GFP + *rol*-*6(su1006)*]		Heat shock inducible body wall muscles	Paraquat hypersensitivity; formation of aggregates under oxidative stress		[101]
	*P*_*snb*-*1*_ *::SOD1(WT,G85R)* -*YFP*	*iwIs8gf*	Constitutive pan-neuronal	G85R and G85R-YFP: severely reduced forward crawling, thrashings and strong resistance to aldicarb. H46R/H48Q-YFP produced a movement defect less prominent than that seen in G85R-YFP		[102]
	N2; *Is* [P_*sng*-*1*_ *::SOD*-*1 (WT, A4* *V, G37R, G93C)*–EGFP]			Increased aggregation formaton; SOD1(G85R) heterodimeric worms have significantly impaired locomotion and reduced lifespan		[103]
	*lin* -*15(n765ts);* [P_*rgef*-*1*_ *::FUS (WT, R514G, R521G, R522G, R524S and P525L)* + P_*pab*-*1*_:: *mcherry; lin*-*15(*+*)*]	*pJH897*		Formation of cytoplasmic FUS aggregates; R522G, P525L, FUS513 and FUS501: significantly shorter lifespan. P525L, FUS513 and FUS501: partially or completely paralysed, severely shrunken by 8 days of age		[104]
	P_*unc*-*54*_::*SOD1(WT, G85R, G93A, G127insTGGGstop)*::YFP	AM263; AM265	Constitutive muscles	Accumulation of mutant SOD1 causes 25–30 % decrease in motility on day 2 of adulthood, and further decrease by approx. 10 % on day 6 of adulthood		[105, 106]
	*unc*-*119(ed3);Is[P*_*unc*-*47*_ *::TDP*-*43*-*(WT, A315T)* + *unc*-*119(*+*)]* *unc*-*119(ed3); Is[P*_*unc*-*47*_ *::FUS*-*(WT, S57Δ)* + *unc*-*119(*+*)]*	*xqIs132, xqIs133, xqIs173, xqIs98*	GABAergic motor neurons	Have normal lifespan, but displayed adult-onset, age-dependent loss of motility, progressive paralysis, neuronal degeneration, accumulation of highly insoluble TDP-43 and FUS proteins	Methylene blue, salubrinal, guanabenz, and phenazine; resveratrol, rolipram, reserpine, trolox, propyl gallate, and ethosuximide	[62]
	[P_*snb*-*1*_ *::TDP*-*43*-*YFP WT(iwIs26)*], [P_*snb*-*1*_ *::TDP*-*C25*-*YFP(iwIs22)*], [P_*snb*-*1*_ *::TDP*-*43*-*YFP Q331* *K(iwEx20)*], [P_*snb*-*1*_ *::TDP*-*43*-*YFP M337* *V(iwEx28)*], [P_*snb*-*1*_ *::SOD1*-*YFP WT(iwIs27)*] and [P_*snb*-*1*_ *::SOD1*-*YFP G85R(iwIs8)*]	IW63, IW33, IW20, IW46, IW31, IW8	Constitutive pan-neuronal	Transgenic models developed robust locomotion defects and protein aggregation		[107]
	P_*unc*-*25*_ *::G93A SOD1*-*GFP*		GABAergic motor neurons	Age-dependent paralysis; G93A SOD1 aggregates in neural cell bodies and causes axon guidance defects		[108]
ALS/FTLD-U	N2; *Is*[P_*snb*-*1*_ *::TDP*-*43 (WT, G290A, A315T, M337* *V)* + P_*snb*-*1*_:GFP]	CK405, CK406, CK410; CK422; CK423; CK426	Constitutive pan-neuronal	Mutant TDP-43: significantly impaired locomotion; degeneration of GABAergic motor neurons	PHA767491; LDN-0130436	[109]
ALS/FTLD-U	*Is*[P_*unc*-*25*_::SNB-1::GFP] + *Ex*[P_*snb*-*1*_:TDP-43; P_*regf*-*1*_:: DsRed2; P_*unc*–*122*_:RFP]	CL2609, CL1681, CL1682		Unc and abnormal motor neuron synapses		[110]
FTDP-17	*N2; Is*[P_*aex*-*3*_:: *4R1* *N human tau (WT, V337* *M, P301L)* + P_*myo*-*2*_ *::GFP*]	CK10, CK49, CK1301, CK1310	Constitutive pan-neuronal	Mutant tau: strong age-dependent progressive uncoordination and accumulation of insoluble tau; neurodegeneration; presynaptic cholinergic transmission defect; reduced lifespan	Azaperone, clofazimine, isoniazid, lorglumide, nefopam, perphenazine, trazodone, zotepine; ethosuximide	[34, 37, 38]
	Pro-aggregant lines: *N2; Is*[P_*rab*-*3*_::*F3ΔK280* + P_*myo*-*2*_::mCherry]	BR5270, BR5485, BR5944, BR5706		Strongly defective locomotion at day 1 of adulthood, accelerated aggregation of insoluble Tau, severe developmental defects of nervous syetem, impaired presynaptic transmission	Methylene blue, BSc3094, bb14 and cmp16	[36]
	Anti-aggregant lines: *N2; Is*[P_*rab*-*3*_::*F3ΔK280(I277P)(I308P)* + P_*myo*-*2*_::mCherry]	BR5271, BR5486, BR6516, BR6427		No obvious locomotion defects and minimum perturbation of the development of the nervous system		
	*N2; Is [*P_*mec*-*7*_::*tau WT(0N4R, 0N3R)* + *rol*-*6(su1006)*]	*tmIs82, tmIs83, tmIs84, tmIs85, tmIs171; tmIs110, tmIs173*	Touch neurons (ALML/R, AVM, PLML/R, PVM); weak in FLP, PVD, BDU	Age-dependent progressive impairment in touch response; neurodegeneration; tau WT4R: little tau accumulation in PLM neuron		[35]
	*N2; Is [*P_*mec*-*7*_:: *tau (P301L, R406* *W)* + *rol*-*6(su1006)*]	*tmIs81, tmIs178, tmIs179; tmIs146, tmIs147, tmIs148, tmIs149*		Strong age-dependent progressive impairment in touch response; neurodegeneration; strong tau accumulation in PLM neuron		
	*pha*-*1(e2123ts)*; *Ex*[P_*rgef*-*1*_::*Tau*_*352*_ *(WT, PHP, Ala10)* + *pha*-*1(*+*)*]	VH255, VH1016, VH1018; VH254, VH1014, VH1015; VH418, VH421	Constitutive pan-neuronal	Both WT and PHP tau_352_ showed age-dependent progressive uncoordination and neurodegeneration; no change in motor neuron viability. Mutant PHP tau: altered motor neuron development. Ala10 tau: early onset of movement defects and reduced lifespan		[33]
HD	P_*unc*-*54*_ *::polyQ*-GFP/YFP/CFP	*pEGFP*-*N1*-*Q19, pEGFP*-*N1*-*Q82*	Constitutive muscles	Length-dependent formation of aggregates; growth rates slowed down; reduced motility	Icariside II; NG-094; aspirin	[9, 111, 112]
	P_*unc*-*54*_ *::DRPLAP*-*Q(32, 40, 56, 79)*-GFP	*pCKX2004, pCKX2003, pCKX2002, pCKX2001*		Q > 40: formation of cytoplasmic aggregates		[113]
	P_*mec*-*3*_ *::htt57Q(19, 88, 128)*-GFP P_*mec*-*3*_ *::htt57Q(19, 88, 128)* ::CFP + P_*mec*-*7*_:YFP	ID24, ID1	Mechanosensory neurons	Highly penetrant posterior touch insensitivity, significant anterior Mec phenotype; significant deposits and morphological abnormalities in PLM cell axons	Resveratrol	[114, 115]
	N2; *rmEx*[P_*rgef*-*1*_ *::HttQ (0,19,35,40,67,86)*-CFP/YFP]	CFP lines: (Q35) AM303; (Q40) AM305; (Q67) AM308; (Q86) AM313. YFP lines: (Q35) AM78 and AM80; (Q40) AM85 and AM87; (Q67) AM81 and AM83; (Q86) AM322 and AM324	Constitutive pan-neuronal	PolyQ length-dependent aggregation; overt neuronal dysfunction; polyQ length-dependent decrease of thrashing, pharyngeal pumping and erratic defecation cycle	β-Lapachone	[40]
	*rtIs11*[P_*osm*-*10*_::GFP + P_*osm*-*10*_::*HttQ150* +*Dpy*-*20*(+)]	HA659	Chemosensory neurons	Severe defect in the nose touch response		[41]
	*pqe*-*1(rt13) III*; *rtIs11*[P_*osm*-*10*_::GFP + P_*osm*-*10*_::*HttQ150* + *Dpy*-*20*(+)]	HA759		Accelerated polyQ mediated neurodegeneration. Vast majority (>90 %) of ASH neurons undergo cell death in less than 3 days	Lithium chloride, mithramycin, trichostatin; rotenone, oligomycin and 2,4-dinitrophenol; *D. officinarum* extracts; salidroside	[42, 43]
	*N2; rmIs[P*_*unc*-*54*_ *::polyQ(0, 24, 35, 37, 40)::YFP]*	(Q35) AM140*; (*Q37) AM470; *(*Q40) AM141	Constitutive muscles	Q35 and Q37 aggregation in muscle cells causes a significant motility defect	AM140: ML346; celecoxib; NT219 AM141: salidroside	[106]
MJD	Full-length ATXN-3 expressing lines: P_*rgef*-*1*_ *::AT3q14, AT3q75, AT3q130::*YFP	AM491, AM513, AM509, AM494, AM519, AM520, AM666, AM685, AM599	Constitutive pan-neuronal	PolyQ length-dependent aggregation and motor dysfunction	17-(allylamino)-17-demethoxygeldanamycin (17-AAG), valproic acid	[116]
	C-terminal ATXN-3 expressing lines: P_*rgef*-*1*_ *::257cAT3q14, 257cAT3q75, 257cAT3q80, 257cAT3q128::*YFP	AM396, AM416, AM422, AM391, AM428, AM419, AM420, AM684, AM683, AM702		Worms with truncated ATXN3 expression have similar aggregation profiles in their neurons and have more severe motility defects		
	N2; [P_*unc*-*54*_ *257cAT3(Q45)*::YFP] or P_*unc*-*54*_ *257cAT3(Q63)*::YFP		Constitutive muscles	PolyQ length-dependent toxicity; aggregation and toxicity are not significantly modulated by aging		[117]
PD	N2; *Is*[P_*unc*-*54*_ *::α*-*syn::*GFP + *rol*-*6(su1006)*]	UA49	Constitutive muscles	α-Syn misfolding and accumulation		[118]
	*Is*[P_*unc*-*54*_ *::α*-*syn::*YFP + *unc*-*119(*+*)*]	NL5901		Formation of inclusions	10-*O*-*trans*-*p*-Coumaroylcatalpol	[119]
	P_*aex*-*3*_ *::α*-*syn (WT, A53T)* + P_*aex*-*3*_::GFP/P_*dat*-*1*_::GFP		Constitutive pan-neuronal	Motility deficits, significant dopaminergic neuron loss and dendritic breaks		[120]
	P_*(acr*-*2, unc*-*30)*_ *::α*-*syn (WT, A53T)* + P_*aex*-*3*_::GFP/P_*dat*-*1*_::GFP		Motor neurons			
	N2; *Is*[P_*unc*-*119*_ *::α*-*syn (WT, A53T, β*-*syn)* + *pDPSU006*-GFP]		Constitutive pan-neuronal	A53T: greater vulnerability to rotenone-induced toxicity, exhibiting 68.4 % lower survival after 4 days of 50 μM rotenone treatment		[53]
	P_*dat*-*1*_ *::α*-*syn (WT, A53T)* + P_*dat*-*1*_::GFP	BY273, UA18, UA31, UA44	Dopaminergic neuron	Mean life span was similar among the non-Tg, WT, and A53T α-synuclein-expressing strains; significant DAergic neuron loss and dendritic breaks	Acetaminophen; bromocriptine and quinpirole; valproic acid; spermidine	[120–124]
	P_*dat*-*1*_ *::α*-*syn (A30P, A53T, A56P)* + *P*_*dat*-*1*_::*mCherry*		Dopaminergic neuron	Increased neurodegeneration; A30P or A53T: failure in modulation of locomotory rate in response to food and markedly reduced DA content (~1 ng/g vs N2 ~5 ng/g). A56P: more impaired in DA-dependent behaviour		[125]
	N2; *Is*[P_*unc*-*51*_ *::α*-*syn (WT, A53T, A30P)* + P_*unc*-*51*_::EGFP]		Constitutive pan-neuronal	No motor deterioration or retardation in growth		[126]
	N2; *Is*[P_*mec*-*7*_ *::α*-*syn (WT, A53T)* + *rol*-*6 (su1006)*]		Mechanosensory neurons	Moderate impairments in touch response		
	P_*unc*-*51*_ *::S129A or S129D α*-*syn* + P_*unc*-*51*_:EGFP P_*unc*-*51*_ *::S129A or S129D α*-*syn* + P_*unc*-*25*_: SNB-1::GFP		Constitutive pan-neuronal	Strikingly severe motor defects throughout development and aging, growth retardation, and synaptic abnormality. SNB-1::GFP fluorescence was broadly diminished in the nerve cord		[127]
	*lin*-*15(n765ts); Is*[P_*snb*-*1*_ *::LRRK2 (WT, G2019S, R1441C, KD, R1441C/KD*) + P_mec-4_::GFP; *lin*-*15 (*+*)*]	*wlzIs1*-*7*	Constitutive pan-neuronal	G2019S LRRK2 increased vulnerability of dopaminergic neurons to mitochondrial stress. Reduced lifespan in mutant LRRK2 (G2019S or R1441C)		[128]
	N2; *baIn20* [P_*dat*-*1*_ *::LRRK2 (G2019S)* + P_*dat*-*1*_ ::GFP]	UA118	Dopaminergic neuron	Age-dependent degeneration of DAergic neurons, behavioural deficit, locomotor dysfunction and depletion of dopamine(~72 % loss). G2019S causes more rapid progression of behavioural deficits than others	GW5074, indoline; sorafenib	[60]
	BY250*; baEx129*[P_*dat*-*1*_::LRRK2(G2019S/D1994A)]	UA215, UA216				
	*lin*-*15(n765ts) X; Is*[P_*dat*-*1*_ *::LRRK2 (WT,R1441C, G2019S, K1347A)* + P_*dat*-*1*_ :GFP + *lin*-*15 (*+*)*]	SGC722, SGC851, SGC856, SGC862			TTT-3002 and LRRK2-IN1	[61, 129]
	*lin*-*15(n765ts) X; cwrEx900* [P_*dat*_-1::GFP, P_*dat*_-1::*LRRK2(R1441C/A2016T), lin*-*15(*+*)*]	SG900, SGC910		Double mutants displayed DAergic defects and neurodegeneration similar to R1441C- and G2019S-LRRK2 models.		[61]
Prion	*lin*-*15(n765ts);* [P_*mec*-*7*_ *::PrP(WT, PG13)* + P_*Str*-*1*_: GFP; *P*_*mec*-*7*_::GFP + *lin*-*15 (*+*)*]		Mechanosensory neurons	Progressive loss of response to touch at the tail caused by mutant (PG13-PrP) PrP expression without causing cell death	Quinacrine, resveratrol	[130]
	P_*ric*-*19*_::PrP + P_*ric*-*19*_::GFP	*cgIs51, cgIs52, cgIs53*	Constitutive pan-neuronal	High PrP levels cause abnormal morphology, striking neuropathogenic phenotypes and remarkable reductions in lifespan		[131]
	*rmIs319*[P_*unc*-*54*_::sup35(rΔ2-5, *nm, r2e2*)::yfp],	AM801, AM803, AM806	Constitutive muscles	Profound cell autonomous and cell non-autonomous disruption of mitochondrial integrity, embryonic and larval arrest, developmental delay, widespread tissue defects, and loss of organismal proteostasis		[132]
Mutant/RNAi						
AD	*apl*-*1(yn10)*			Larval lethality, defects in molting and morphogenesis		[133]
	*apl*-*1(*RNAi)			Reduced body size, with some worms exhibiting L4 molting problems		[99]
	*sel*-*12(ar131)* and *(ar171)*	GS1894		Exhibit thermotaxis defects		[134, 135]
ANCL	*dnj*-*14(ok237)* *dnj*-*14(tm3223)*	RM2754 TM3223		Age-dependent progressive impairment in locomotion, severe progressive chemosensory defects which precede neurodegeneration of sensory neurons and significantly shorter lifespan	Resveratrol, rolipram; ethosuximide	[38, 71]
PD	*lrk*-*1(km17), (km41), (tm1898) and (RNAi)*			Mitochondrial stress, ER stress sensitive		[128]
	*pdr*-*1(lg103), (XY1046, Parkin KO3) and (RNAi)*			Display severe developmental defects and lethality at early larval stages in presence of ER stressors. Majority died or arrested at, or prior to, the larval L3 stage. 15.4 % shorter life span than that of non-Tg strain		[53, 136]
	*pink*-*1(tm1779)*			Increased sensitivity to a 3-day exposure to 150 mM paraquat		[137]
	*djr*-*1.1*(RNAi)			Significantly more sensitive to rotenone treatment than control nematodes		[53]
SMA	*smn*-*1(ok355) I/hT2*[*bli*-*4(e937) let*-*?(q782) qIs48*] *(I;III)*	LM99		Thrashing rate progressively declined and almost completely ceased after 5 days post-L1. Pharyngeal pumping rates showed a rapid and progressive decline. Mean lifespan is 6.0 vs 17.7 days for N2	Riluzole	[138]
	*smn*-*1(cb131)I*	LL2073		Body length and lifespan was significantly shorter than that of the WT; defective motility, egg-laying and hatching	4-Aminopyridine, gaboxadol hydrochloride, N-acetylneuraminic acid	[77]
Chemical treatment						
PD	*vtIs7*[P_*dat*-*1*_::GFP] subjected to 6-hydroxydopamine (6-OHDA)	BY250, BY200		Neuronal process blebbing, cell body rounding with process loss and cell body loss reproducibly appear in this order within a few hours	Bromocriptine, quinpirole and memantine; acetaminophen; Chondrus crispus extract	[122, 139–141]
	N2; [P_*cat*-*2*_::GFP], *egIs1*[P_*dat*-*1*_::GFP] subjected to 1-methyl-4-phenyl-1,2,3,6-tetrahydropyridine (MPTP)	BZ555		Reduced mobility, increased lethality and DA neurodegeneration	Lisuride, apomorphine and rottlerin; P7C3, P7C3A20; polysaccharides from *Chaenomeles speciosa;* acetylcorynoline; *n*-butylidenephthalide	[54]
	N2; [P_*dat*-*1*_::α-syn + P_*dat*-*1*_::GFP] subjected to Manganese (Mn^2+^)			Oxidative stress, mitochondrial stress, enhanced DA neurodegeneration, reduced DA levels		[121]
	*pink*-*1(tm1779)* subjected to Paraquat			Oxidative stress		[137]
	*pdr*-*1(XY1046),* P_*snb*-*1*_ *::α*-*syn WT,* P_*unc*-*119*_ *::α*-*syn A53T*, N2, *lrk*-*1(km17),* P_*snb*-*1*_ *::LRRK2 (WT, R1441C, G2019S)* subjected to Rotenone			Mitochondrial stress, reduced viability	D-α-hydroxybutyrate in combination with tauroursodeoxycholic acid	[53, 128]
	P_*dat*-*1*_::GFP subjected to *Streptomyces venezuelae* secondary metabolite			DA neurodegeneration		[142]

Human neuorodegenerative diseases (NDs): *AD* Alzheimer’s disease, *ANCL* adult-onset neuronal ceroid lipofuscinosis, *ALS* amyotrophic lateral sclerosis, *CJD* Creutzfeldt-Jakob disease, *FTDP-17* Frontotemporal dementia with parkinsonism-17, *FTLD-U* frontotemporal lobar degeneration with ubiquitinated inclusions, *HD* Huntington’s disease, *MJD* Machado–Joseph disease (or spinocerebellar ataxia type 3), *PD* Parkinson’s disease, *SMA* spinal muscular atrophy

### Caenorhabditis elegans as a model for human neurodegenerative disease

A major challenge to the identification of effective disease-modifying therapies arises from an insufficient knowledge about the contribution of multiple pathways to disease pathogenesis. Mammalian disease models offer in vivo opportunities and extensive similarity to the human brain, but testing the therapeutic value of small molecules in mammalian model systems is extremely expensive and requires time-consuming experimental designs that can be prohibitive. Over the past decades, *C. elegans* has increasingly been used as a model system to study the underlying molecular mechanisms that give rise to neurodegeneration because of its well-characterised and easily accessible nervous system, short generation time (≈3 days) and lifespan (≈3 weeks), tractability to genetic manipulation, distinctive behavioural and neuropathological defects, coupled with a surprisingly high degree of biochemical conservation compared to humans. Remarkable similarities exist at the molecular and cellular levels between nematode and vertebrate neurons. For example, ion channels, receptors, classic neurotransmitters [acetylcholine, glutamate, γ-aminobutyric acid (GABA), serotonin, and dopamine (DA)], vesicular transporters and the neurotransmitter release machinery are similar in both structure and function between vertebrates and *C. elegans* [5, 6]. Importantly, the impact of different challenges such as genetic perturbations or exposure to drugs on the survival and function of defined neuronal populations in the *C. elegans* nervous system can be readily studied in vivo.

To date, various laboratories have developed and characterised a diverse set of *C. elegans* models of various human NDs, including AD [7], PD [8] and polyglutamine expansion diseases [9] (Table 1). These worm ND models have been developed by over-expressing human ND-associated genes (both wild type and mutant versions) and by mutating or altering the expression level of the orthologous worm genes. Strong parallels were especially observed in the genotype-to-phenotype correlations between the human NDs and the phenotypes of transgenic *C. elegans* ND models. This supports the validity of the approach as expression of mutant human proteins in *C. elegans* can closely model a fundamental property of these mutations in humans.

Nevertheless, there are also limitations to using *C. elegans* to model NDs that must be considered. Although the worm offers huge potential for experimental manipulations, there are aspects of ND pathophysiology that cannot easily be modelled in worms. For example, abundant evidence supports an important role for brain inflammation and microglial cell activation in several NDs, notably AD [10], but there is no microglial equivalent among the 56 glial cells of *C. elegans*. Clearly, the very simplicity of the worm nervous system that makes it so attractive for studying basic neurobiology is also a disadvantage in that the complexity of the mammalian brain cannot be adequately reflected, and so rodent models will continue to be required to validate any findings from *C. elegans* ND studies. There are also potential pitfalls of using *C. elegans* for drug screening, as many compounds do not easily penetrate the worm’s protective cuticle [11] and as biotransformation of compounds by the worms’ *E. coli* food source may give misleading pharmacological information [12]. Although these potential pitfalls can be mitigated by combining predictive bioaccumulation algorithms [11] with increased dose regimens, and by confirming drug effects using metabolically inactive *E. coli*, these issues need to be considered when performing drug screens in worms.

**Fig. 1:**
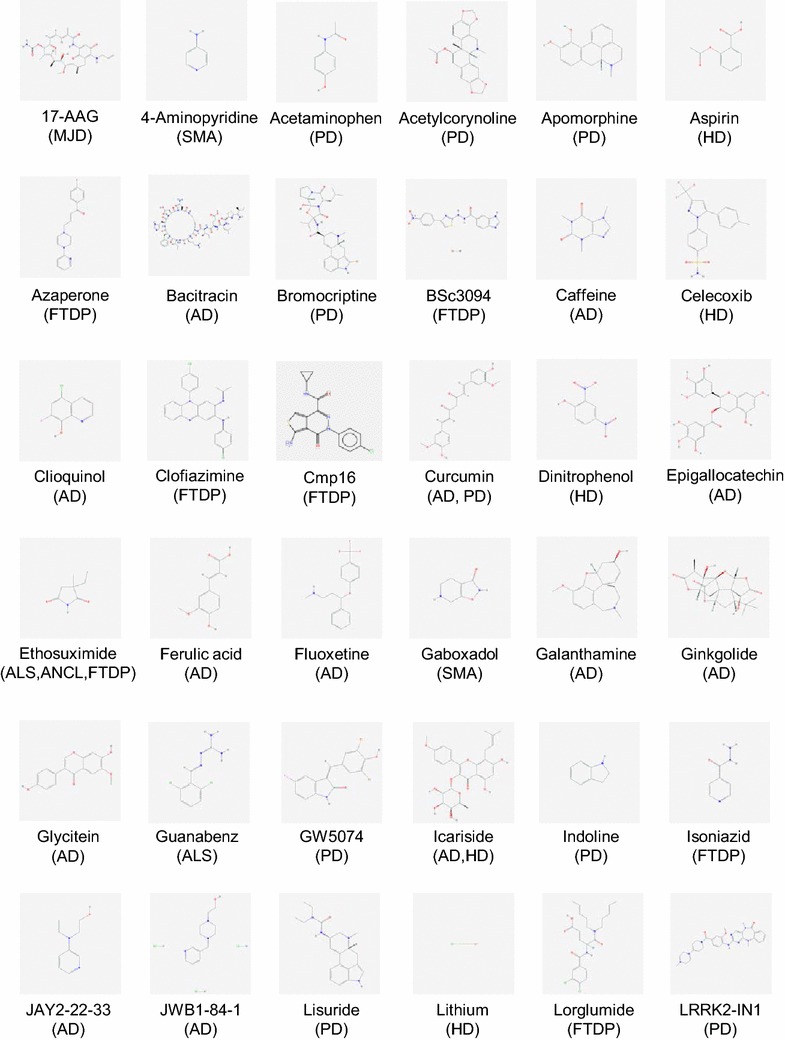
Structures of compounds with therapeutic effects in *C. elegans* models of human neurodegenerative diseases. Chemical structures were obtained from PubChem (https://pubchem.ncbi.nlm.nih.gov) or MolBase (http://www.molbase.com). *AD* Alzheimer’s disease, *ALS* amyotrophic lateral sclerosis, *ANCL* adult-onset neuronal ceroid lipofuscinosis, *FTDP* frontotemporal dementia with parkinsonism-17, *HD* Huntington’s disease, *MJD* Machado–Joseph disease (spinocerebellar ataxia type 3), *PD* Parkinson’s disease, *Prion* prion disease, *SMA* spinal muscular atrophy

**Fig. 2:**
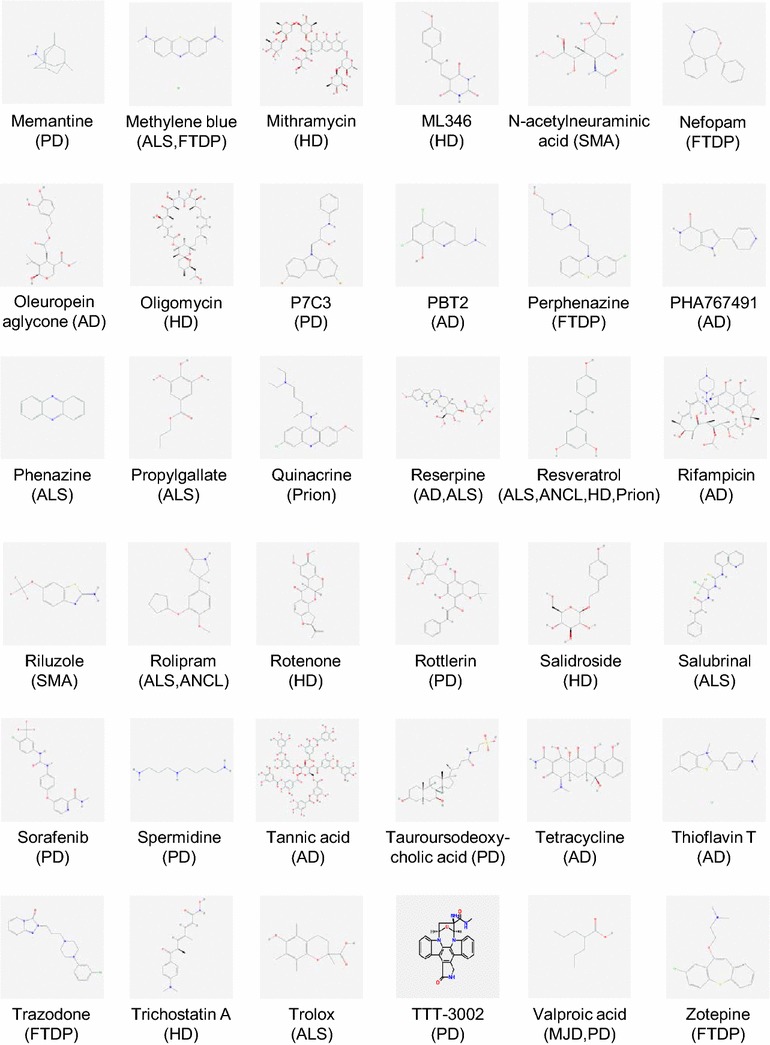
Structures of compounds with therapeutic effects in *C. elegans* models of human neurodegenerative diseases. Chemical structures were obtained from PubChem (https://pubchem.ncbi.nlm.nih.gov) or MolBase (http://www.molbase.com). *AD* Alzheimer’s disease, *ALS* amyotrophic lateral sclerosis, *ANCL* adult-onset neuronal ceroid lipofuscinosis, *FTDP* frontotemporal dementia with parkinsonism-17, *HD* Huntington’s disease, *MJD* Machado–Joseph disease (spinocerebellar ataxia type 3), *PD* Parkinson’s disease, *Prion* prion disease, *SMA* spinal muscular atrophy

Despite the above caveats, *C. elegans* remains a widely used animal model to identify genes that modify neurodegeneration in vivo. Indeed, genetic screens performed on worm models have identified a wide variety of conserved genes that can suppress or increase disease progression and are thus potential therapeutic drug targets. However, relatively few of these genetic modifiers are common to more than one disease model, despite the shared feature of protein misfolding/aggregation [13, 14]. In addition to its utility for screening for genetic contributors to NDs, *C. elegans* is a useful pharmacological model for testing potential neuroprotective compounds. Numerous well-characterised ND models have been readily exploited for triaging compounds from large libraries consisting of novel and pre-approved drugs, and for testing the effects of individual drugs, prior to validation in vertebrate models. Potential therapeutics identified via such compound screens using specific worm ND models are shown in Figs. 1, 2, listed in Table 1 and described in detail below.

#### Alzheimer’s disease: amyloid-β (Aβ) models

β-Amyloid is the main component of the extracellular plaques found in the brains of Alzheimer’s disease patients. It is widely (though not universally) believed that aggregation of Aβ into oligomeric forms is the main driver of neurodegeneration in Alzheimer’s disease. This has been modelled in nematodes by expressing human Aβ constructs in worm muscle cells [7]. The Aβ-induced paralysis observed in the well-characterised muscle-specific strains has provided a valuable phenotype for straightforward quantification of the effects of treatments on Aβ toxicity and validation of potential therapeutic interventions for Alzheimer’s disease. The *C. elegans* strain CL2006, which constitutively expresses human Aβ_1-42_, has been elegantly used to demonstrate the neuroprotective effects of a diverse range of compounds (Table 1; Figs. 1, 2). These include natural products such as specific gingkolides [15], soya isoflavone glycitein [16], the green tea component epigallocatechin gallate [17, 18] and coffee extract [19]; FDA-approved drugs such as tannic acid, bacitracin, rifampicin [20], thioflavin T [21], reserpine [22] and the antidepressant fluoxetine; and polyphenolic compounds such as curcumin and ferulic acid [23, 24]. These treatments conferred considerable life-span extension and cellular stress tolerance [15, 16]. This was a consequence of most compounds attenuating the rate of toxic human Aβ_1–42_ mediated paralysis, to suppress the Aβ_1–42_ induced increase in toxic reactive oxygen species and hydrogen peroxide levels, and to inhibit Aβ_1–42_ oligomerisation and deposition [15, 25]. Recent studies have also demonstrated how the antibiotic tetracycline and its analogues [26], and ethanol extract of Liuwei Dihuang [27] successfully protected the CL4176 inducible Aβ_1–42_ muscle-specific expression model by inhibiting Aβ_1–42_ oligomerisation and reducing superoxide production. Oleuropein aglycone, the main polyphenol in extra virgin olive oil, was recently shown to protect against amyloid toxicity in both constitutive and inducible Aβ_1–42_ models [28]. In addition, two recent large, unbiased yeast-based screens of pharmacological modifiers identified the 8-hydroxyquinoline chemical scaffold (8-OHQ), a class of clinically relevant bioactive metal chelators as neuroprotective compounds that reduced proteotoxicity associated with the aggregation of several ND-specific proteins including TDP-43, α-synuclein, polyglutamine proteins, or Aβ_1–42_ [29, 30]. Notably, two closely related 8-OHQs–PBT2 and clioquinol, which conferred neuroprotective benefits in mouse models of AD, were further shown to rescue Aβ_1–42_ toxicity in *C. elegans* body wall muscle cells [31] and glutamatergic neurons [30]. PBT2 was also effective in improving cognition and reducing Aβ in cerebrospinal fluid in a small Phase IIA trial in AD patients [31].

#### Tauopathies

**Fig. 3:**
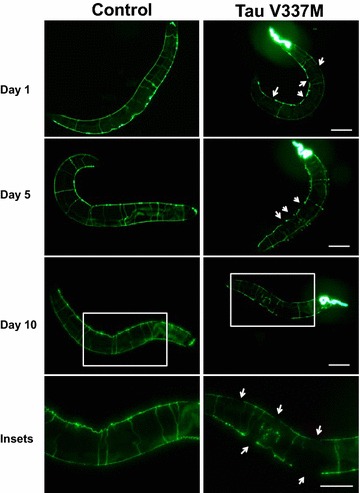
A *C. elegans* genetic model of the Tauopathy, FTDP-17. Triple transgenic worms expressing human V337M mutant Tau protein (Paex-3::V337M Tau), a pharyngeal GFP marker (Pmyo-2::GFP) and a GFP reporter transgene marking the cell bodies and processes of all *C. elegans* GABAergic neurons (Punc-25::GFP) were compared with control single Punc-25::GFP transgenic worms. All *panels* are micrographs of representative whole worms. Control (*left panels*) and Tau V337M expressing worms (*right panel*) were examined after 1, 5 and 10 days of age. In control worms, intact ventral and dorsal cords were observed at all ages. In contrast, the mutant Tau transgenic GABAergic reporter strain exhibited severe degeneration of neuronal processes. Ventral and dorsal cord gaps (*arrows*) are disruptions in the continuity of the ventral and dorsal nerve cords, respectively. *Scale bar* represents 200 μm for all *panels* except for the *bottom two panels*, which are high magnifications of the *boxed areas* of day-10 worms shown above

In addition to amyloid plaque deposition, Alzheimer’s disease is associated with intraneuronal accumulation of neurofibrillary tangles containing the microtubule-associated protein Tau, which aggregates into insoluble fibrillar deposits when it is hyperphosphorylated [32]. Pathological Tau deposits are also observed in Pick’s disease, corticobasal degeneration, Down’s syndrome and specific types of frontotemporal dementia (FTD) such as frontotemporal dementia with parkinsonism chromosome 17 type (FTDP-17) and frontotemporal lobar dementia (FTLD). Various worm transgenic Tauopathy models expressing mutant human Tau constructs have therefore been generated and yielded complementary findings in regards to the effects of neuronal Tau expression [33–35]. Neurodegeneration in worms expressing transgenic human mutant Tau can be assessed indirectly, using phenotypes such as impaired locomotion and reduced lifespan, but also directly by visualising loss of neuronal cell bodies and neuronal processes in vivo. An example of the latter is shown in Fig. 3, where a human Tau construct containing the FTDP-17-associated V337 M mutation is expressed in all 302 worm neurons via a pan-neuronal *C. elegans* promoter. In addition, the 26 GABAergic neurons of the worm are specifically labelled by driving green fluorescent protein (GFP) expression from GABA-specific *C. elegans* promoter. In control worms, a continuous, intact line of GFP fluorescence is seen running along both the ventral and dorsal nerve cords on opposite sides of the animal. In contrast, the mutant Tau transgenic strains exhibits large gaps in these nerve cords where neuronal processes are missing, thus directly demonstrating severe neurodegeneration in the living animal.

Using such Tauopathy models, compounds with known anti-aggregation activity like methylene blue, were shown to effectively ameliorate the worms’ motility and neuronal defects [36]. In addition, a novel compound belonging to the aminothienopyridazine class, cmp16, was also shown to rescue these phenotypes and to suppress Tau aggregation in worms [36]. Importantly, aminothienopyridazines are known to suppress Tau aggregation in mammalian cells and so the improved blood–brain barrier permeability of cmp16 suggests that this compound may have significant translational potential. In a recent screen of a library of FDA-approved compounds, dopamine D2 receptor antagonism was identified as a promising strategy for targeting tau-induced neurotoxicity, as antipsychotics such as azaperone, perphenazine, and zotepine improved the phenotypic features of Tauopathy in worms (Table 1; Figs. 1, 2). Azaperone, in particular, effectively ameliorated mutant Tau-induced functional defects and reduced the level of insoluble Tau aggregation [37]. Finally, a recent study reported that the anti-epileptic drug, ethosuximide, could ameliorate the impaired motility and reduced lifespan phenotypes of the Tau V337 M worm FTDP-17 model [38]. Interestingly, ethosuximide’s action in this worm Tau model was independent of its main proposed target in epilepsy, the T-type calcium channel.

#### Polyglutamine (polyQ) disorders

Expansion of trinucleotide CAG repeats in a variety of different genes leads to neurodegenerative diseases such as Huntington’s disease and spinocerebellar ataxias due to the expression of a polyglutamine tract within the encoded protein. Diverse worm transgenic models where varying lengths of polyQ tracts are expressed in specific sets of neurons, muscle cells and even intestine cells have been widely used to model several aspects of polyQ neurotoxicity, notably to address the mechanisms underlying the impact of aggregation prone proteins on cellular function and to identify novel disease modifiers [39–41]. The progressive nature of polyQ-mediated toxicity, protein aggregation and general severity of phenotype demonstrated in these models is age- and polyQ-tract-length-dependent, recapitulating critical aspects of polyglutamine expansion diseases in patients.

Voisine et al. [42] screened candidate pharmacological compounds utilising a HD model in which the *pqe*-*1* genetic mutant background greatly enhanced toxicity induced by a human Huntingtin construct containing a 150-residue glutamine tract (Htt-Q150). Both lithium chloride and mithramycin alleviated neuronal cell death, while trichostatin A (a class I and class II HDAC inhibitor) provided significant neuroprotection. Using the same HD model, Varma et al. [43] discovered that small molecular inhibitors of metabolism (mitochondrial and glycolytic function) such as rotenone, oligomycin and 4-dinitrophenol rescued neuronal loss and degeneration by activating caspase inhibition and ERK and AKT prosurvival signalling and their efficacy was further validated in cell culture and *Drosophila* HD models (Table 1; Figs. 1, 2). Resveratrol, a demonstrated activator of sirtuin deacetylases, also effectively alleviated Htt-Q128 toxicity in both worm and neuronal culture models [44]. Recently, treatment of a *C. elegans* model of SCA3 (spinocerebellar ataxia type 3; also known as Machado-Joseph disease) with 17-(allylamino)-17-demethoxygeldanamycin (17-AAG), an HSP90 inhibitor, successfully decreased the mutant ATXN3 aggregation and improved locomotor activity [39]. Treatment of the same model with valproic acid (VA), another HDAC inhibitor and a well-known anti-epileptic drug, also led to improved locomotor activity accompanied by a decrease in mutant ATXN3 aggregation. Therefore, HDAC inhibitors which promote histone acetylation over deacetylation and which were also known to provide protection against polyQ mediated toxicity in vertebrate and *Drosophila* neurons may hold promise as a preventive therapy in polyQ diseases.

Other pan-neuronal or neuron specific HD models facilitated the identification of other potential therapeutic interventions, including the anti-cancer agent β-lapachone [45], *D. officinarum* root extracts [46] and a phenol glycoside salidroside [47], which conferred protection against polyQ neuronal toxicity. Treating *C. elegans* muscle polyQ models with hydroxylamine, icariin and celecoxib derivatives (NG-094, icariside II and OSU-03012, respectively) ameliorated polyQ-mediated protein aggregation and protected against polyQ proteotoxicity [48–50] (Table 1; Figs. 1, 2). Aspirin, an analgesic agent, was also shown to significantly improve polyQ-mediated animal paralysis, reducing the number of Q35-YFP aggregates and delaying polyQ-dependent acceleration of aging [51].

#### Parkinson’s disease (PD)

**Fig. 4:**
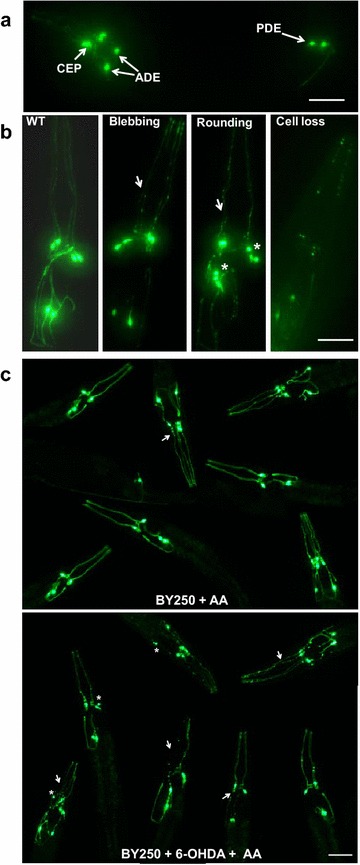
A *C. elegans* model of toxin-induced Parkinson’s disease. **a** Dopaminergic (DA) neuronal cell bodies and neurites in BY250 worms were visualised using an integrated Pdat-1::GFP dopamine transporter marker. *C. elegans* has eight DA neurons: six are located in the anterior region, which can be subclassified in pairs as two anterior deirid neurons (ADE), two dorsal cephalic neurons (CEP) which are postsynaptic to the ADE neurons and two ventral CEPs that are not postsynaptic to the ADEs; two posterior deirid neurons (PDE) located posteriorly are also shown. *Arrows* depict the four CEP neuron processes and indicate the ADE and PDE cell bodies in a young worm. Anterior is to the *left*. **b** Representative examples of worms scored which display the three characteristic stages of DA neurodegeneration in response to 6-OHDA. Magnification of anterior region of C. elegans shows only the anterior-most DA neurons. WT: in this example, all six anterior DA neurons of this worm appear robust and the dendrites are intact and fully extended. Neuronal process blebbing; cell body rounding: this worm exhibited prominent cell body rounding (*asterisk*) and dendrite blebbing (*arrows*); cell body loss: this worm exhibited a complete loss of GFP in most DA neurons as CEP and ADE neurons have all degenerated and are no longer visible in any focal plane, only retention of GFP expression in the remnants of neuron cell bodies and broken neurites. All *scale bars* represent 20 μm. **c** Representative images of worms 24 h post-6-OHDA-exposure are presented. BY250 worms treated with ascorbic acid (AA) alone expressed intact and strong GFP in all six DA neurons and dendrites in the heads. However, the majority of BY250 worms incubated with 50 mM 6-OHDA showed a marked GFP expression reduction in the dendrites of ADEs and CEPs, many of the cell somas became round (*asterisk*) and blebs appeared along the dendrites of CEPs (*arrows*)

Pathologically, PD is characterised by degeneration of dopaminergic neurons in the substantia nigra and accumulation of Lewy bodies containing aggregated α-synuclein protein. Although most cases are idiopathic, PD can be caused by both environmental (e.g. pesticide exposure) and genetic (e.g. α-synuclein and LRRK2 mutation) effects. Multiple worm PD models, notably the toxin-induced models, have aided in the discovery and validation of potential pharmacological interventions for PD. An example of how dopaminergic neurodegeneration can be directly assessed in vivo in *C. elegans* is shown in Fig. 4. Here, the eight dopaminergic neurons of the worm are specifically labelled by GFP expression from the promoter of the *C. elegans* dopamine transporter. In control worms, fluorescent neuronal cell bodies extending long processes are clearly visible in the head (6 neurons) and tail (2 neurons) of the animal. However, treatment with the PD-inducing toxin, 6-hydroxydopamine (6-OHDA), causes the loss of GFP-labelled dopaminergic neuronal cell bodies and/or processes, thus enabling direct visualisation of neurodegeneration.

Chemical screens have suggested that compounds which protect mitochondria or increase autophagy protect against α-synuclein toxicity [52, 53]. Braungart et al. [54] performed a focused compound screen using the *C. elegans* MPTP model of PD and found that lisuride and apomorphine (dopamine receptor agonists), as well as rottlerin (protein kinase C inhibitor) ameliorated the MPTP-induced behavioural defects when present at a low concentration. In addition, nomifensine (dopamine transporter inhibitor), nicotine (acetylcholine receptor agonist), selegiline (monoamine oxidase inhibitor), MPEP (mGluR-5 inhibitor), amantadine, α-lipoic acid (antioxidant) and ascorbic acid (antioxidant) were effective at higher concentrations [53]. In another screen, two mammalian dopamine D2 receptor agonists, bromocriptine and quinpirole, were identified to confer significant neuroprotection independent of dopamine receptors in a 6-OHDA-induced dopaminergic neurodegeneration model of PD [55]. Similarly, a low concentration of acetaminophen (analgesic and antipyretic) was reported by Locke et al. [56] to protect significantly against 6-OHDA toxicity-induced dopaminergic neurodegeneration in P_*dat*-*1*_::GFP expressing worms. However, the protection appears to be selective as acetaminophen was not neuroprotective against α-synuclein-induced neurodegeneration at any concentration tested. The anti-epileptic drug, valproic acid provided significant dopaminergic neuroprotection in a *C. elegans* PD model associated with human α-synuclein overproduction, which was further shown to be mediated through ERK-MAPK signalling [57]. A more recent study has also demonstrated the neuroprotective effects of the naturally occurring polyamine spermidine and phytocompounds such as *n*-butylidenephthalide, curcumin, N-acetylcysteine and vitamin E on 6-OHDA-induced degeneration of dopaminergic neurons and their ability to attenuate α-synuclein accumulation. *n*-butylidenephthalide, in particular, had the greatest neuroprotective capacity and was shown to also restore food-sensing behaviour and dopamine levels in both pharmacological and transgenic *C. elegans* PD models as well as enhancing the life span of 6-OHDA-treated animals [58]. Acetylcorynoline, the major alkaloid component derived from *Corydalis bungeana*, a traditional Chinese medical herb demonstrated the same neuroprotective effects when applied to the same pharmacological and transgenic *C. elegans* PD models [59].

Kinase-targeted inhibition of LRRK2 protein activity was recently established as an effective treatment for PD as LRRK2 kinase inhibitors consistently mitigated pathogenesis caused by different LRRK2 mutations. Liu et al. [60] showed that though GW5074, an indoline compound, and sorafenib, a Raf kinase inhibitor, did not have protective effects against α-synuclein- and 6-OHDA-induced toxicity, they increased survival and reduced dopaminergic neurodegeneration in G2019S-LRRK2 transgenic *C. elegans* and *Drosophila*. Yao et al. [61] further demonstrated the potency of kinase inhibitors as they were able to pharmacologically rescue both the behavioural deficit and neurodegeneration manifested by the expression of mutant LRRK2 G2019S and R1441C in vivo using two LRRK2 inhibitors, TTT-3002 and LRRK2-IN1, which also potently inhibited in vitro kinase activities of LRRK2 wild-type, R1441C and G2019S at nanomolar to low micromolar concentrations when administered either pre-symptomatically or post-symptomatically. Compounds that have been shown to be protective in the various worm PD models are listed in Table 1 and their chemical structures shown in Figs. 1 and 2.

#### Amyotrophic lateral sclerosis (ALS)

A number of transgenic lines expressing mutant forms of human SOD1 found in familial ALS patients under a range of promoters have been generated and recapitulated the motor neuron degeneration and paralysis characteristic of ALS patients [102, 103, 105, 108]. Genes recently shown to be mutated in ALS include the DNA/RNA binding proteins *TDP*-*43* and *FUS*, and *C9ORF72,* a novel familial and sporadic ALS causative gene. Treatment with methylene blue, an aggregation inhibitor of the phenothiazine class, not only rescued toxic phenotypes (including neuronal dysfunction and oxidative stress) associated with mutant TDP-43 and FUS in *C. elegans* and zebrafish ALS models [62], but also ameliorated Tau mediated toxicity in a newly established *C. elegans* model [36]. Using transgenic TDP-43 models, Tauffenberger et al. evaluated 11 compounds previously reported to enhance longevity in *C. elegan*s and resveratrol (polyphenol), rolipram (phosphodiesterase 4 inhibitor), reserpine (antihypertensive), ethosuximide (anti-epileptic), trolox and propyl gallate (antioxidants) were revealed as effective candidates that protected against mutant TDP-43 toxicity in motor neurons [63] (Table 1; Figs. 1, 2). Recent genetic experiments by Kraemer’s group suggested that inhibiting cell division cycle kinase 7 (CDC7) kinase activity reduces phosphorylation of TDP-43 and the consequent neurodegeneration. Small molecule inhibition of CDC-7 by PHA767491 was further shown to robustly reduce TDP-43 phosphorylation and prevent TDP-43 dependent neurodegeneration both in vitro and in vivo [64].

#### Autosomal dominant adult-onset neuronal ceroid lipofuscinosis (ANCL)

ANCL, also known as autosomal dominant Kufs’ disease and Parry disease, is a rare hereditary disease characterised by intra-neuronal inclusions of autofluorescent lipofuscin-like material and neurodegeneration [65, 66]. Recently, four independent research groups have reported that ANCL is caused by mutations in the *DNAJC5* gene that encodes the endogenous neuroprotective synaptic chaperone cysteine string protein (CSP) [67–70]. Our lab has recently developed a *C. elegans* model of ANCL by using null mutants of the worm *DNAJC5* orthologue, *dnj*-*14* [71]. These worms have similar phenotypes to ANCL patients and also to CSP mutants in mice, in terms of reduced lifespan, progressive neuronal dysfunction and neurodegeneration [72]. This evolutionary conservation of CSP’s neuroprotective function suggests that the worm *dnj*-*14* model could have potential for identifying generic neuroprotective interventions rather than disease specific drug targets. Indeed, a focused screen of pharmacological compounds that ameliorated the *dnj*-*14* lifespan and neuronal defects identified the polyphenolic molecule resveratrol, which has been shown to be neuroprotective in a range of animal neurodegeneration models [71]. In contrast to other worm neurodegeneration models [44, 63, 73, 74], however, resveratrol acted in a *sir*-*2.1*-independent manner, as *sir*-*2.1; dnj*-*14* double mutants showed full lifespan rescue by resveratrol. Instead, the mechanism of resveratrol action appeared to be via inhibition of cAMP phosphodiesterase, as the phosphodiesterase inhibitor, rolipram was shown to mimic the effect of resveratrol in rescuing *dnj*-*14* phenotypes [71]. More recently, the anti-epileptic drug ethosuximide has been shown to be protective in the *dnj*-*14* model, acting through a DAF-16/FOXO-dependent mechanism that is distinct from its proposed mechanism of action in epilepsy [38]. Ethosuximide also ameliorates the phenotypes of worm models of FTDP-17 [38] and ALS [63] and reduces protein aggregation in a mouse neuronal cell culture model of Huntington’s disease [38], suggesting that it may have general and evolutionarily conserved neuroprotective properties. Indeed, it has recently been shown that ethosuximide reverses cognitive decline in a rat model of Alzheimer’s disease [75]. Finally, a recent genome-wide transcriptional profiling study of *dnj*-*14* mutants revealed a striking reduction in expression of ubiquitin proteasome system (UPS)-related genes in comparison to wild type control strains [76]. Genes encoding components of multimeric E3 ubiquitin ligases were especially over-represented, suggesting that these may represent potential novel drug targets for treatment of ANCL and perhaps other neurodegenerative diseases.

## Translational implications of *C. elegans* chemical screens

The different screening strategies that have been applied to *C. elegans* ND models have provided distinct insights into potential therapeutic approaches in patients. These strategies range from robotic automated imaging-based approaches designed for high throughput compound library screening [77] to highly focused screens of a selected small group of compounds that target a common pathological process such as protein aggregation [21]. Large scale screens offer greater coverage of chemical space and so have potential to identify unifying pharmacological themes amongst multiple hits from compound libraries. For example, several different dopamine D2 receptor antagonists were recovered as hits in an unbiased library screen using a Tauopathy model, with genetic techniques then being used to confirm that reduced D2 receptor function is indeed neuroprotective [37]. Whilst this suggests that several currently prescribed atypical anti-psychotic drugs could potentially be re-purposed for treatment of human tauopathies, dosing regimens would need to be carefully considered given reports that the relatively high doses of these medications used to treat aggression and agitation in dementia patients may increase the risk of death [78].

One observation that emerges from our analysis of the large number of studies to date is that very few compounds are therapeutic in multiple *C. elegans* ND models. Indeed, out of the 72 compounds shown in Figs. 1 and 2, only ethosuximide and resveratrol are effective in more than two ND models and therefore appear to have general neuroprotective activity. This may be due in part to the fact that most published studies have focused on relatively small sets of compounds and so activity across multiple ND models remains to be tested. Nevertheless, it seems certain that this also reflects disease-specific pharmacological actions—for example, Raf kinase inhibition is therapeutic in LRRK2-based PD models, but ineffective in α-synuclein- and 6-OHDA-based PD models [60]. Clearly, effective clinical treatments with such highly disease-specific drugs requires knowledge of the underlying pathophysiological mechanism, which is not always diagnosable in NDs. Drugs such as ethosuximide and resveratrol are therefore potentially very useful, as they may provide general neuroprotective activity regardless of uncertainties regarding molecular pathology. The mechanism of action of ethosuximide and resveratrol remains unclear and controversial [79–81], but both have been linked to increased longevity and healthspan in model organisms [82, 83]. Given that dietary restriction, the best established intervention known to increase longevity and healthspan, is therapeutic in multiple ND models from invertebrates to mice [84], it is clear that slowing the ageing process can confer general neuroprotection. It may be that ethosuximide and resveratrol modulate some of the same conserved neuroprotective mechanisms that decline with age, thus potentially explaining their therapeutic effects in radically different ND models.

## Conclusions and future perspectives

The nematode *C. elegans* has great potential for expediting neuroprotective drug discovery. Its facile genetics and suitability for high-throughput compound screening mean that both target-driven and phenotypic screening approaches can easily be performed (and potentially combined). Although phenotypic screening became unfashionable as a drug discovery paradigm in the post-genomic era, Swinney and Anthony have clearly shown that most new medicines still continue to be discovered via phenotypic screening [85]. This influential work has forced a re-evaluation in the pharma industry and a consequent shift towards phenotypic screening that incorporates available knowledge of targets/mechanisms [86], for which *C. elegans* is ideally suited. Furthermore, there is increasing evidence that using compound combinations designed to act on multiple molecular targets can be an effective therapeutic strategy—as exemplified by the spectacular success of combination therapy for HIV [87]. Testing of many such drug combinations can be performed rapidly and cheaply using worm models, in contrast to rodent models. In addition, technical developments such as CRISPR [88] now offer the potential to rapidly create new and more accurate *C. elegans* models of human neurodegenerative diseases, by precisely delivering single-copies of mutant genes identified from patients to appropriate desired locations in the worm genome. Although *C. elegans* has already facilitated the identification of potential novel therapeutics, the future combination of more accurate genetic models with high-throughput automated drug screening platforms is a potentially very efficient strategy for therapeutic drug discovery for NDs.
